# Stakeholder Perspectives of Implementation Barriers of Artificial Intelligence in Eye Care: A qualitative framework-based study

**DOI:** 10.1007/s44402-026-00080-w

**Published:** 2026-04-13

**Authors:** Judy Nam, Angelica Ly, Sarita Herse, Chris Lim, Mary-Anne Williams, Fiona Stapleton

**Affiliations:** 1 School of Optometry and Vision Science, UNSW Sydney, Sydney, Australia; 2 School of Management and Governance, UNSW Sydney, Sydney, Australia; 3 Department of Ophthalmology, National University, Singapore, Singapore; 4 Center for Sustainable Medicine, Yong Loo Lin School of Medicine, National University, Singapore, Singapore; 5 Singapore Eye Research Institute, Singapore, Singapore; 6 AI Institute, UNSW Sydney, Sydney, Australia

**Keywords:** Artificial intelligence, Barriers, Clinical decision support systems, Implementation, Stakeholders

## Abstract

**Purpose:**

Despite the revolution of artificial intelligence (AI), its integration remains limited in healthcare. A comprehensive understanding of the barriers to implementation is crucial to enhance the utilisation of AI. This study applies a conceptual framework-based analysis, to explore stakeholder perspectives of implementation barriers of AI in digital diagnosis in eye care.

**Methods:**

Purposive sampling was used to identify key individuals across stakeholder groups, including technology developers, clinicians, patients and healthcare leaders. Semi-structured interviews were conducted with 37 stakeholders. Using the updated Consolidated Framework for Implementation Research (CFIR), responses to the question: ‘What is the biggest barrier to digital diagnosis or AI for macular disease in Australia?’ were analysed. Barriers identified by stakeholders were mapped to thematic constructs of the updated CFIR, and the prominence of each implementation barrier was measured. Data saturation was not assessed.

**Results:**

For clinicians and developers, the ‘innovation’ domain was most frequently cited. Clinicians were most concerned with the costs involved, whereas for developers, a lack of evidence surrounding real-world application was the main challenge. For leaders and patients, ‘individuals’ domain was the most frequently cited. Leaders were focused on the innovation deliverers: expressing the potential risk of over-reliance on the innovation and the subsequent consequence of clinician deskilling. Patients were more concerned about innovation recipients: emphasising the perceived lack of human empathy with the implementation of AI.

**Conclusions:**

Differences were revealed in the identified barriers to the implementation of AI across stakeholder groups. A co-design approach to address the misalignment in key barriers may be essential to the successful implementation of AI in digital health innovations.

Key Points

- Applications of artificial intelligence hold the potential to transform eye care, but its integration in current practice remains limited.
- Stakeholder analysis reveals the perceived barriers to the implementation of artificial intelligence in digital diagnosis across groups of developers, clinicians, patients and healthcare leaders in eye care.
- While there are some shared barriers, a fundamental misalignment in perspectives highlights the importance of co-design in guiding successful artificial intelligence implementation into the future.

## Introduction

Artificial intelligence (AI) is transforming everyday lives with its ability to simulate human intelligent behaviour [1] across a wide range of applications [2]. It is becoming an increasingly popular tool utilised across various fields, including healthcare [3], where it can improve diagnostic accuracy and quality of care [4]. As AI can enhance clinical decision-making [5, 6], there has been a growing interest in augmenting its use for digital diagnosis and improving patient outcomes [4] across disciplines.

In eye care, AI can aid in preventing avoidable blindness and vision impairment. One key example of its potential to revolutionise current clinical practice is in diagnosing and managing age-related macular degeneration (AMD). AMD is a leading cause of vision loss with a global prevalence of 8.7% [7] and is a growing public health concern as it is frequently underdiagnosed and misdiagnosed in several healthcare settings [8, 9]. This leads to delayed management and vision-threatening consequences, as the progression of AMD is characterised by central vision loss. Furthermore, AMD has two presentations known as ‘dry’ (non-neovascular) or ‘wet’ (neovascular) AMD, which is important in order to differentiate to provide appropriate management for the patient. AI can be applied to any stage of the patient journey, from improving early detection and diagnosis to management and triaging for treatment [10, 11].

However, it is evident from the literature that despite the significant potential of AI to transform eyecare, its integration remains limited across healthcare [12]. Although there are known challenges to the general implementation of AI, including cost, liability and data privacy [13–15], the real-world barriers to its application in digital diagnostic tools in eye care, specifically AMD, have not been explored.

This study used qualitative interviews with key stakeholders to explore the barriers to adopting AI in macular disease and the factors contributing to its lack of clinical utilisation in diagnosis. Recognising a co-design approach is fundamental to ensuring both the success and broad uptake of these technologies, clinicians, patients, healthcare leaders and technology developers were interviewed to capture their diverse perspectives. For analysis, the updated CFIR [16]—a well-established determinant framework in implementation science to evaluate contextual factors influencing real-world outcomes and overall implementation effectiveness, was used. The purpose was to identify key factors that could hinder the effective implementation of AI-driven diagnosis in eye care.

## Methods

### Study Design

A qualitative study based on data from a series of semi-structured interviews with key stakeholders (including clinicians, developers, leaders and patients) was conducted by researchers at the UNSW Sydney School of Optometry and Vision Science and School of Business (Supplementary file 1).

#### Participant Selection

##### Sampling

Purposive sampling was used to identify 37 key individuals from the professional contact list of authors to have a diverse representation across stakeholder groups (12 clinicians, 10 healthcare leaders, eight patients with AMD and seven developers). All participants were 18 years o or older and had an interest and/or experience in macular disease.

##### Method of Approach (Recruitment) and Sample Size

From September 2022 to March 2023, 147 potential participants were contacted by email. Participation was voluntary and no financial incentives were offered for the completion of the study.

Informed consent was obtained from all participants in accordance with the Declaration of Helsinki and approved by the University of New South Wales, Sydney Human Research Ethics Advisory Committee (HC210986; February 2022).

#### Data Collection

Demographic data, including age, sex and occupation, were obtained using an online questionnaire (Qualtrics, qualtrics.com). All interviews were conducted in English via Zoom (Zoom Communications Inc., zoom.com) by authors AL and SH [17].

##### Interview Guide

A semi-structured interview guide was developed by research investigators following a literature review to explore key questions for each stakeholder group [17]. Questions were designed to encourage open dialogue of AI and diagnostic technologies for AMD in Australia and were structured in sections: (1) experiences, (2) attitudes, (3) enablers, (4) barriers, (5) possible futures (anticipated personal and business use). Interviewers were encouraged to reword, re-order or clarify questions to facilitate the discussions.

Participants were provided with a copy of the guide before their interview and were asked to complete the interview only once. All participants were informed that the purpose of the interview was to hear their experiences, views and attitudes toward digital diagnosis for macular disease, especially neovascular AMD.

##### Audio Recording and Transcription

All interviews were audio-recorded, transcribed verbatim and analysed using a theory-driven approach based on the updated CFIR [16] in NVivo (Lumivero, lumivero.com). The updated CFIR enabled systematic analysis of interview data and assessment of contextual factors influencing real-world implementation.

##### Definitions

Definitions have been adapted from the updated Consolidated Framework for Implementation Research (CFIR) [16], with the elimination of sub-domains that were not identified (Table 1).  AMD, age-related macular degeneration.

**Table 1:** Adapted CFIR and definitions.

**I. Innovation domain** The “thing” being implemented *Project innovation*: Artificial intelligence and related technologies for the digital diagnosis of AMD, especially neovascular AMD, which remain in practice when implementation is complete and are distinct from the implementation process and strategies used to implement the technology.	
**Construct name**	**Construct definition** *The degree to which:*
A. Innovation Source	The group that developed and/or visibly sponsored use of the innovation is reputable, credible and/or trustable.
B. Innovation Evidence-Base	The innovation has robust evidence supporting its effectiveness.
C. Innovation Relative Advantage	The innovation is better than other available innovations or current practice i.e., better than a human clinician’s ability to diagnose AMD
D. Innovation Adaptability	The innovation can be modified, tailored, or refined to fit local context or needs.
E. Innovation Complexity	The innovation is complicated, which may be reflected by its scope and/or the nature and number of connections and steps.
F. Innovation Cost	The innovation purchase and operating costs are affordable.
**II. Outer setting domain** The setting in which the inner setting exists. There may be multiple outer settings and/or multiple levels within the outer setting. *Project outer setting(s)*: Community and local health district systems (public and private), state-wide health systems, national health schemes (within Australia) and other companies, including affiliations, organisations and referral networks. Boundary between the outer and inner setting is the physical premises of healthcare clinics, bounded by brick and mortar.	
**Construct name**	**Construct definition** *The degree to which:*
A. Critical Incidents	Large-scale and/or unanticipated events disrupt implementation and/or delivery of the innovation.
B. Local Attitudes	Sociocultural values (e.g., shared responsibility in helping recipients) and beliefs (e.g., convictions about the worthiness of recipients) encourage the outer setting to support implementation and/or delivery of the innovation.
C. Partnerships & Connections	Networks with external entities, including referral networks, academic affiliations and professional organisation networks.
D. Financing	Funding from external entities (e.g., grants, reimbursement) is available to implement and/or deliver the innovation.
E. External Pressure	External pressures drive implementation and/or delivery of the innovation.
**II. Inner setting domain** The setting in which the innovation is implemented. There may be multiple inner settings and/or multiple levels within the inner setting. *Project inner setting(s)*: Single or multi-site clinics united by a single brand.	
**Construct name**	**Construct definition** *The degree to which:*
A. Structural Characteristics—Information Technology Infrastructure	Technological systems for tele-communication, electronic documentation and data storage, management, reporting and analysis support the functional performance of the inner setting.
B. Culture	There are shared values, beliefs and norms across the inner setting.
C. Access to Knowledge & Information	Guidance and/or training is accessible to implement and deliver the innovation.
**IV. Individuals domain** The roles and characteristics of individuals. *Project role(s)*: Innovation deliverers and recipients. *Project characteristic(s)*: Need, capability and motivation of individuals’ roles.	
**Construct name—roles**	**Construct definition** *The degree to which:*
A. Innovation Deliverers	Individuals who are directly or indirectly delivering the innovation—clinicians, developers and leaders.
B. Innovation Recipients	Individuals who are directly or indirectly receiving the innovation – patients.
**Construct name—characteristics**	**Construct definition** *The degree to which:*
1. Need	The individual(s) has deficits related to survival, well-being, or personal fulfilment, which will be addressed by implementation and/or delivery of the innovation.
2. Capability	The individual(s) has interpersonal competence, knowledge and skills to fulfil Role.
3. Motivation	The individual(s) is committed to fulfilling role.
**V. Implementation process domain** The activities and strategies used to implement the innovation. *Project implementation process*: Dynamic sustainability framework; activities and strategies.	
**Construct name—roles**	**Construct definition** *The degree to which:*
A. Assessing Needs—Innovation Recipients	Collects information about the priorities, preferences and needs of recipients to guide the implementation and delivery of the innovation.

#### Data Analysis

##### Data Coding

AL extracted participants’ responses to the question *‘What is the biggest barrier to digital diagnosis or AI for macular disease in Australia?*’. Other relevant remarks, including the keyword ‘barrier’, were also extracted for the analysis. Two authors AL and JN annotated the extracted text segments of the transcripts with the specific barrier to AI adoption using an inductive approach, with no predefined barrier list.

Although each participant was encouraged to describe the single biggest barrier to implementation, if more than one was mentioned, it was counted separately. If the same barrier was mentioned more than once by a participant during their interview, it was only counted once.

Once all the barriers had been summarised, iterative meetings were performed to review coded texts and cross-check results. Agreement was reached on 65 of all 72 identified barriers, translating to an inter-coder reliability of 90%. Disagreements were discussed by AL and JN to arrive at an agreement on the optimal concept to code a specific fragment of the text after checking the full transcript context and re-reading the section of interest. Where ambiguity remained, the CFIR framework definitions were consulted to guide coding decisions. The usual sources of disagreement were the scope of one concept (3/7) and the specific barriers and facilitators that one concept should encompass (4/7). In the latter cases, a single concept was split into two separate concepts until agreement was achieved.

The derived index of specific barriers was subsequently mapped to the constructs of the updated CFIR. Any disagreements surrounding the most appropriate updated CFIR construct were resolved by discussing the possible options until an agreement was reached. All authors verified that the domains and sub-domains appropriately described the extracted data.

##### Assessment of Key Barriers

To quantify the prominence of the barrier to implementation, the frequency of each cited barrier coded under each domain and sub-domain across stakeholders was compared (Table 2). CFIR, Consolidated Framework for Implementation Research.

**Table 2:** Barrier counts.

CFIR domain	CFIR sub-domain		Specific barriers	Clinicians *n* = 12	Developers *n* = 7	Leaders *n* = 10	Patients *n* = 8	Totals *n* = 37
Innovation	Innovation Source		Limited credibility of the innovation source	0	1	0	0	1
	Innovation Evidence-Base		Lack of evidence and uncertainty about whether sufficient resources are available to prove clinical usefulness.	2	2	1	0	5
	Innovation Relative Advantage		Generalisability: The innovation seemed inaccurate when applied locally and did not improve the clinical workflow.	4	1	0	0	5
	Innovation Adaptability		Interoperability: Limited capacity to work with different imaging instruments, algorithms and software.	2	1	1	0	4
	Innovation Complexity		Nature of ocular imaging being difficult to acquire clear images	0	1	0	0	1
	Innovation Cost		Purchase and operating costs	5	1	1	0	7
				13	7	3	0	23
Outer setting	Critical Incidents		Hackers obtaining personal health information	1	0	0	0	1
	Local Attitudes		Uncertainty about whether it would be possible to maintain equity across different organisations and patient populations	0	1	3	0	4
	Partnerships & Connections		Lack of partnerships and connections to support utilisation	1	0	0	0	1
	Financing		Uncertainty about financing; role of government subsidy	0	2	1	1	4
	External Pressure		Misaligned priorities of different stakeholders	0	0	1	0	1
				2	3	5	1	11
Inner setting	Structural Characteristics —Information Technology Infrastructure		Lack of infrastructure to ensure appropriate data security and privacy; fear of data misuse	2	1	0	0	3
			Large data storage requirements	2	0	0	0	2
	Culture		Resistance to change	2	1	1	1	5
	Access To Knowledge & Information		Optometrists’ lack of training in being able to use the AI recommendations	1	2	1	0	4
				7	4	2	1	14
Individuals	Innovation Deliverers	Need	Optometrists’ fear of job loss	0	0	1	0	1
		Capability	Risk of over-reliance	1	1	3	0	5
			Fear that the professional judgement of clinicians will be diminished	0	0	1	0	1
		Motivation	Lack of trust in the technology itself	1	1	1	0	3
	Innovation Recipients	Need	Patient need for human empathy and oversight	2	0	1	4	7
		Motivation	Lack of individual patient awareness regarding the need for eye care	1	1	0	1	3
				5	3	7	5	21
Implementation process	Assessing Needs —Innovation Deliverers		Training and knowledge of innovation deliverers, a persistent need to retrain	1	0	2	0	3
				1	0	2	0	3

## Results

A total of 37 individuals across four stakeholder groups participated in the study. Of the participants, there were 12 clinicians (32%), 10 healthcare leaders, including senior health managers and professional organisation representatives (27%), eight patients with AMD (22%) and seven developers, including researchers or technologists (19%). Stakeholder groups were labelled as clinicians (C), developers (D), leaders (L) and patients (P).

Despite the interview guide stating that the purpose was to explore AI and related technologies for the digital diagnosis of macular disease, specifically neovascular AMD, participants were more focused on the innovation aspect (i.e., AI) during the interview. The ensuing discussions were not limited to macular disease but covered the implementation of digital diagnosis in eye health care in general.

Table 3 summarises the domain count across the stakeholder groups in order of highest to lowest frequency. For clinicians and developers, ‘innovation’ was the most frequently quoted domain. For leaders and patients ‘individuals’ were the most cited; however, leaders focused on innovation deliverers, whereas patients were more concerned about innovation recipients. Implementation processes were mentioned the least across the stakeholders as an identified barrier.

**Table 3:** Key domains identified by stakeholder groups.

Clinicians	Developers	Leaders	Patients
Innovation (13)	Innovation (7)	Individuals (7)	Individuals (5)
Inner setting (7)	Inner setting (4), Individuals (4)	Outer setting (5)	Outer setting (1), Inner setting (1)
Individuals (5)		Innovation (4)	
Outer setting (2)	Outer setting (3)	Inner setting (2), Implementation processes (2)	
Implementation process (1)			

### Clinicians

**Fig. 1:**
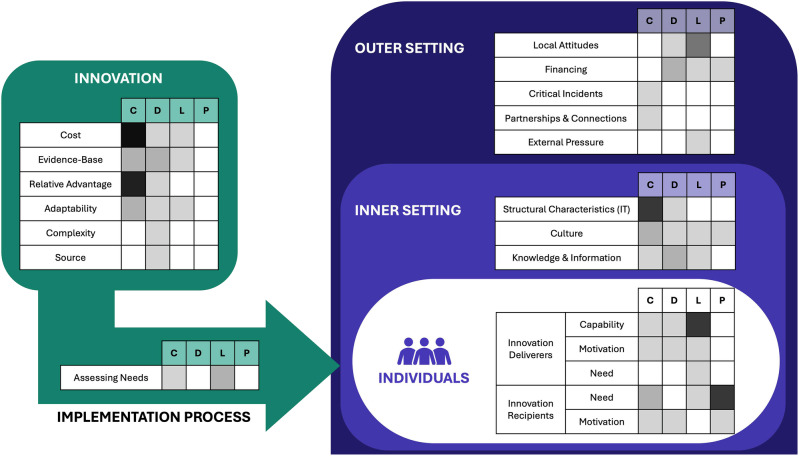
Barriers mapped across domains and stakeholder types: clinicians (C), developers (D), leaders (L) and patients (P). Adapted from The Center for Implementation’s image [60] of the updated Consolidated Framework for Implementation Research (CFIR) [16].

The costs of purchasing and operating an innovation were expressed by many clinicians as a key barrier to its implementation (Fig. 1).

*“The only barrier for practitioners to acquire it is cost…The only barrier for every practice to have it…is cost”*—C4

*“To be honest, the biggest factor…is cost…assuming it works reasonably well, I think, it’s mainly a cost thing.”*—C12

Clinicians also cited concerns of generalisability and the innovations’ lack of relative advantage over their own ability to make accurate diagnoses (Fig. 1).

*“If the AI incorrectly does, like pick up an artefact…an error in judgement can be made…it highlights that there’s something severely wrong and they’re unnecessarily referred [false positive] or the opposite where it misses something and they’re told…there’s nothing wrong [false negative].”*—C8

The structural limitations within their clinical practices were addressed (Fig. 1), with the lack of information technology infrastructure to support the functionality of the innovation, including data storage and cybersecurity (digital health privacy).

*“We are still some ways away from having the infrastructure to do so…hackers managed to obtain personal information”*—C1

*“Other barriers…data storage, privacy issues”*—C12

### Developers

Developers identified similar barriers to clinicians correlating to the innovation domain.

“*I think people have issues with AI and decision support…how can I trust that system has done it the same way that I would have as a clinician?*”—D6

“*It comes back to knowledge, right?…Like how do they know this is legit? That whatever technology that is put forward to me is safe, is accurate, is relevant?”*—D5

However, their views were more generalised across subdomains (Fig. 1); with the only barrier with repeated citations being the lack of evidence for the innovation and hence the uncertainty of its clinical usefulness in the real world.

*“I think it’s definitely…a near term issue in terms of getting enough data to make that really work”*—D1

*“So that’s a real barrier for us, conducting the research itself in a setting…out into the world to do, you know, pragmatic studies, but that’s not currently what we’re doing yet.”*—D2

### Leaders

On the other hand, leaders focussed on highlighting the potential risk of over-reliance on the innovation and the consequent deskilling of clinicians (Fig. 1).

*“AI lends itself to sort of protocol-based care…you feed this information in, you get a green light or a red light…but I think the risk is over-reliance on that…people just sort of blindly trusting it”*—L1

*“People get…a bit blasé about re-checking because it’s quite good for a while and then there’s a false negative and they’re not doing the adequate other tests…but it doesn’t have the same sensitivity or specificity”*. – L3

*“It’s an algorithm, it’s a model….but people in the system have got to remember that that’s what it is. It’s a tool. It’s an aid…So people have to be aware that you can’t over-rely on a model…Practitioners need to appreciate both the strength of an AI diagnosis as well as the shortcomings and where the tool might be at its weakest. So I think that worries me because people have a tendency to over-rely on technology these days.”*—L5

Another commonly expressed barrier was the uncertainty of being able to maintain equity across different organisations and patient populations (Fig. 1).

*“It’s also about how you roll out the accessibility and availability of these tools to the broader community”*—L5

*“One of the problems across the health area is consistency. And so you want everybody to get the same level of care…You’re building up a new silo with people who are for digital health… and people who are not so knowledgeable”*—L7

### Patients

Patients posed the most individual-centric outlook (Fig. 1), with the key barrier being the need and the lack of human empathy with the implementation of AI.

*“I think there’s sort of an added element in the personal connection…the patient if they feel safe and secure, is much more able to speak about their concerns….if you just leave them up to a computer, the computer can only do what it’s set up to do.”*—P5

*“I’m reassured by the fact there is human intervention.”*—P6

*“I like the personal touch…maybe I’m a bit old fashioned still…whereby I’d like to actually be able to ask the person whatever questions…it’s about having the person in the room with you”*—P8

Supplementary file 2 summarises all the other barriers.

## Discussion

Despite the surge in AI development, its practical integration within healthcare remains limited [12]. The present study addressed this by exploring barriers to the implementation of AI for digital diagnosis in eye care and found that clinicians are concerned about the cost and financing of AI, developers face challenges proving its usefulness in the clinical workflow, leaders foresee the consequences of clinicians' deskilling due to potential over-reliance on AI and patients have apprehension for the absence of human interaction.

Most importantly, these results highlight the differing focus of each stakeholder group and their perceived barriers, whereby misalignment of perspectives can be an inherent barrier to implementation. Barriers to AI in healthcare are typically explored from the perspective of clinicians [18], leaders [19] and patients [20] or an incomplete combination of stakeholders [21, 22]. To our knowledge, there is currently no framework-based analysis encompassing all the stakeholders’ perspectives. Stakeholder co-design is crucial from the initial development phase [23, 24] of digital health innovations to deliver patient-centred care successfully [24]. Thus, a synthesised evaluation (Table 4) combining multiple stakeholder perspectives is fundamental to achieving a more holistic understanding of barriers to AI in digital diagnosis.

**Table 4:** Summary of high-frequency barriers (≥5 across all stakeholders), with each theme mapped to its corresponding specific barrier in Table 2 and the Consolidated Framework for Implementation Research (CFIR) construct.

		Clinicians		Developers		Leaders		Patients	
Costs	Financing	•		•	•	•	•		•
Lack of Clinical Usefulness		•		•		•			
Not Improving Workflow		•		•					
Deskilling of Clinicians		•		•		•			
Lack of Human Interaction for Patients		•				•		•	
Resistance to Change		•		•		•		•	

*Costs* refers to “Purchase and operating costs” (Innovation → Innovation Cost). *Financing* reflects “Uncertainty about financing; role of government subsidy” (outer setting → Financing). *Lack of Clinical Usefulness* corresponds to “Lack of evidence and uncertainty whether sufficient resources are available to prove clinical usefulness” (Innovation → Innovation Evidence-Base). *Not Improving Workflow* relates to the generalisability barrier (Innovation → Innovation Relative Advantage). *Deskilling of Clinicians* synthesises “Risk of over-reliance” (Individuals → Innovation Deliverers: Capability). *Lack of Human Interaction for Patients* reflects “Patient need for human empathy and oversight” (Individuals → Innovation Recipients: Need). *Resistance to Change* (inner setting → Culture).

*The count of ‘Costs’ and ‘Financing’ was added as commonly cited together. Each dot represents a frequency of one or more within the stakeholder group.

By default, in the design of the present study, all the stakeholders had at least a fundamental awareness of AI. However, the level of knowledge in the potential role and use cases of digital diagnostic innovations varied across individuals. This acted as both a strength, allowing meaningful contribution to the discussion of barriers, but also a limitation, as their heterogeneous understanding could shape what they considered a “barrier”. These insights further highlight the importance of co-design and the need for establishing a common language and understanding of the technological innovation as a foundation for successful implementation.

### Costs and Financing

The multi-factorial challenge of costs (Innovation) and financing (outer setting) involved in implementing AI digital diagnosis systems was unanimously identified as a key barrier across stakeholder groups, including high initial start-up costs, operating expenses and the uncertainty of financing the implementation of the innovation. These issues are widely cited in the literature [15, 25–27] and highlight the importance of favourable financial models and reimbursement policies in the adoption of innovative technologies such as AI, despite its potential benefits. Further exploration of stakeholders’ willingness to pay [28] can elicit their perceived value and preferences regarding the implementation of AI and contribute towards building a sustainable financial model to overcome the barrier of costs and financing.

### Lack of Clinical Usefulness and Not Improving Workflow (Innovation)

Another inherent barrier arises from the uncertainty of the usefulness of the innovation and whether it ultimately improves clinical workflow. This barrier combines notions of needing robust evidence supporting AI effectiveness, accuracy, ability to outperform clinicians and its lack thereof being a barrier for all innovation deliverers. Initial concerns emerged from the limited number of randomised controlled trials available in the clinical context [29]. Concerns persist due to ongoing lack of generalisability and practicality [30], despite there being an expanding interest in AI. The need for more comprehensive research to minimise publication bias and to prioritise patient-relevant outcomes when evaluating AI in clinical practice has been recognised [30]. Identifying clinical “pain points” for more solution-driven AI and enhancing collaborations between stakeholders [29] are also acknowledged as potential solutions to improving its usefulness and the overall workflow.

### Deskilling of Clinicians

All innovation deliverers expressed the potential risk of over-reliance by clinicians as a barrier, as well as the consequence of deskilling, compromising one’s competence, knowledge and skills. Over-reliance is identified as a precursor to deskilling [25, 31], with concerns at present regarding increased dependence on the capabilities of automation [25]. Deskilling in the clinical context can be defined as a *“situation where clinicians experience reduced opportunities to exercise particular skills”* [32] as an unintended consequence of AI implementation in the medical profession. This is considered an inevitable process in improving efficiency and reducing costs, as exemplified by the now widespread adoption of electronic health records and clinical practice guidelines [33]. The alternative phenomenon of upskilling or reskilling [32, 34], involving enhancing one’s existing skill set or learning new skills, respectively, was not discussed by any of the stakeholders. However, it is crucial to acknowledge that there is a growing consensus that “AI is not going to replace humans, but humans with AI are going to replace humans without AI”[35]. Thus, focusing on the positive potentials of AI, reskilling efforts should centre on preparing eye care professionals to collaborate effectively with AI systems [34] whilst maintaining the clinician’s critical role in patient outcomes.

### Lack of Human Interaction for Patients

Moreover, the lack of human interaction for patients was identified as a key barrier by both innovation deliverers and recipients, but most commonly by the patients. This highlights the significance of the patient-clinician relationship, whereby building rapport is crucial for the patient to entrust the clinician to act on behalf of their health and have their best interests at heart [32]. Thus, this barrier arises from the fear that healthcare is shifting away from patient interactions and towards data analytics, with a loss of the holistic approach [31]. Despite the capability of AI to outperform humans [36], it is clear that patients prefer human interaction over automation [31, 37]. The strive for patient-centred care, which involves patients as active participants in their care, is considered an essential part of high-quality healthcare systems [38]. Thus, co-design of the implementation of AI should be considered such that it can instead foster the connection between clinicians and patients [39].

### Resistance to Change (Culture)

Finally, similar views emerged from all stakeholders, with resistance to change being cited as a barrier by at least one stakeholder in each group (Table 4). Change resistance [40, 41], being an intrinsic and complex problem, is amplified by the sociotechnical implications of AI [42]; with apprehensions raised across healthcare organisations by clinicians [43], patients [44] and healthcare leaders [26] alike. However, resistance to change can be expressed differently amongst the stakeholders [42]. This can take the form of: 1. rejection (disinclination to adopt the innovation, often due to a reluctance to change the status quo), 2. postponement (accepting the innovation in principle but deciding not to adopt it) and/or 3. opposition (actively engaging in strategies to prevent the innovation’s success) [45]. Given the intricate nuances of change resistance, the successful implementation of innovations requires effective change management strategies [46] whereby leaders play a crucial role [47]. Successful navigation of change resistance can be exemplified by the national implementation of eye health electronic patient records in Scotland and Wales, with increasing adoption across England [48–50].

### Strengths and Limitations

The main strength of the present analysis lies in using purposive sampling to identify key stakeholders, which enabled the capture of diverse and informed perspectives on the barriers to implementing AI in eye care. Selecting individuals who play a critical role in the implementation process ensured the insights gathered were comprehensive and reflective of real-world challenges. While the sample distribution across stakeholder groups was uneven, this was by design and reflects real-world variability in end-users and their associated willingness to participate. The use of purposive sampling via the professional contact list of authors, however, introduces selection bias and possible over-representation of AI-engaged participants, though it also ensured the inclusion of key informants with relevant experience in AI and AMD, which influenced the specific number and role of the participants [17]. The uneven group sizes limited the opportunity for comparisons.

This study was conducted within the Australian healthcare system and is AMD-anchored but offers general insights into stakeholder perspectives of AI implementation in eye care. The transferability of these insights to other diseases, demographics, or international settings may be limited by differences in healthcare infrastructure, funding models and disease-specific workflows.

However, the open dialogue format during the semi-structured interviews naturally allowed responses to explore a wider perspective on AI’s role in eye care diagnosis. Notions around cost and financing and deskilling of clinicians are likely to be applicable across ophthalmic diseases and digital diagnosis.

Sustainable reimbursement is well-recognised as a pivotal enabler for the adoption of novel technologies within healthcare systems [15, 51, 52]. Thus, it is critical to understand the stakeholders that determine reimbursement and consider which models, including fee-for-service and value-based care, may best address the financial sustainability of AI implementation in eye care [51]. Recent evaluations of cost-effectiveness and real-world implementation feasibility highlight the importance of aligning AI integration with performance-based reimbursement frameworks; therefore, advocating for hybrid models that incentivise innovation whilst ensuring fiscal sustainability [51, 52].

Another similarity that is echoed in the literature is the need for clinicians to be AI competent. Beyond the imperative for eye care professionals to reskill to utilise AI systems [34], emerging perspectives suggest that AI needs to be integrated at the education level, across different aspects of the clinical curriculum, to combine technical skills with ethical reasoning [15, 53, 54]. Thus, current and future clinicians require adequate training to be equipped to navigate the complexities of AI-driven healthcare [54].

### Recommendations for Future Directions

To reduce barriers to adoption, financing models could be adapted through national procurement guidelines, targeted grants and expanded tax incentives, particularly for small and medium-sized enterprises developing AI tools, as recommended in the National Policy Roadmap for AI in Healthcare [55]. Successful examples, such as Singapore’s semi-automated diabetic retinopathy screening programme, demonstrate how cost savings and clinical efficiency can be achieved [56].

However, cost-effectiveness varies by geography, deployment strategy and healthcare funding model. High-income countries may benefit more due to higher human grader costs, but studies from lower-income settings show mixed results [56]. Fee-for-service, bundled payments and subscription-based deployment strategies are likely to influence uptake. Moreover, tailoring financial strategies to the structure of healthcare systems, whether public, private, or hybrid, can enhance relevance and sustainability. In Australia, corporate-led rollout models have been described as a strong catalyst for uptake, with even a small number of providers able to shift practice norms across the profession [17].

To mitigate deskilling and promote upskilling or reskilling, leaders should proactively redesign training frameworks to preserve core clinical competencies while fostering human–AI collaboration. Strategies include maintaining hands-on learning opportunities, integrating AI literacy into curricula and using AI as a tool for feedback and simulation-based training. Successful examples include double-reading workflows where clinicians assess cases independently before reviewing AI outputs and structured validation sessions that reinforce clinical reasoning [57, 58]. These approaches align with broader calls for safeguards against skill erosion and support a shift toward hybrid intelligence, where clinicians and AI systems co-evolve to enhance diagnostic quality and professional resilience.

The effectiveness of co-design in healthcare highlights its potential to address both resistance to change and concerns about loss of human connection. An initiative in Australia co-designed AI workflows to analyse patient-reported experience measures, enabling clinicians to integrate narrative feedback into quality improvement cycles while maintaining human oversight and accountability [59]. This exemplifies how co-design can preserve the human elements of care, enhance trust and tailor AI systems to clinical realities.

## Conclusion

This framework-based analysis of barriers to implementation of AI in digital diagnosis provides a more complete overview of stakeholder perspectives, highlighting differences in the views of clinicians, healthcare leaders, patients and developers, including researchers or technologists. Five key barriers were identified as costs and financing, lack of perceived clinical usefulness or not improving workflow, deskilling of clinicians, lack of human interaction for patients and resistance to change.

## Supplementary information

Supplementary file 1

Supplementary file 2

## Data Availability

The data and material generated and analysed during the study are not publicly available to protect participant privacy.
