# Supplementary material for: Stakeholder Perspectives of Implementation Barriers of Artificial Intelligence in Eye Care: A qualitative framework-based study
Source: Ophthalmic Physiol Opt. 2026 Apr 13;46(3):449–58. doi: 10.1007/s44402-026-00080-w (PMC13369640; doi:10.1007/s44402-026-00080-w)
Supplement: Supplementary file 1 [file 44402_2026_80_MOESM1_ESM.docx]

**Supplementary file 1.** Consolidated criteria for reporting qualitative studies (COREQ): 32-item checklist.

Developed from Tong A, Sainsbury P, Craig J. Consolidated criteria for reporting qualitative research (COREQ): a 32-item checklist for interviews and focus groups. Int J Qual Health Care 2007; 19: 349-357.

| **No** | **Item** | **Guide questions/description** | **Response** |
| --- | --- | --- | --- |
| **Domain 1: Research team and reflexivity** | | | |
| *Personal Characteristics* | | | |
| 1 | Interviewer/  facilitator | Which author/s conducted the interview or focus group? | AL and SH |
| 2 | Credentials | What were the researcher's credentials? E.g. PhD, MD | JN: MClinOptom, Bsc (Vision) AL: PhD, GradCertOcTher, BOptom(Hons), FAAO SH: PhD, BPsych(Hons) CL: PhD, MMed (Ophthalmology), MD, BMed, BSc (Med) (Hons 1), FAMS MAW: PhD, LL.M., FAAAI, FTSE, FACS, MSc (Computer Science) FS: PhD, FTSE, FAAO, AO |
| 3 | Occupation | What was their occupation at the time of the study? | JN: PhD Candidate  AL: Senior Lecturer SH: Postdoctoral research fellow CL: Lecturer MAW: Professor FS: Scientia Professor |
| 4 | Gender | Was the researcher male or female? | JN: Female AL: Female SH: Female CL: Male MAW: Female FS: Female |
| 5 | Experience and training | What experience or training did the researcher have? | JN is an optometrist with prior qualitative research experience. AL is an optometrist with prior qualitative research experience.  SH is a psychology graduate with experience in social robotics. CL is an ophthalmologist with prior qualitative research experience. MAW is a professor with extensive experience in business innovation and artificial intelligence.  FS is an optometrist with extensive experience in clinical research and qualitative research methods. |
| *Relationship with participants* | | | |
| 6 | Relationship established | Was a relationship established prior to study commencement? | Recruitment used the professional contact lists of the authors and in some cases a pre-existing relationship was present prior to study commencement. |
| 7 | Participant knowledge of the interviewer | What did the participants know about the researcher? e.g. personal goals, reasons for doing the research | Some participants had prior knowledge of AL as a clinician-researcher, and FS and MAW as academic professors. |
| 8 | Interviewer characteristics | What characteristics were reported about the interviewer/facilitator? e.g. Bias, assumptions, reasons and interests in the research topic | JN is an optometrist and PhD candidate. AL and FS are optometrists and research academics at UNSW involved with professional organisations in Australia. CL is an ophthalmologist and academic in Singapore, affiliated with UNSW. SH and MAW are affiliates of the UNSW Business School and AI institute which could have been a source of bias. No other interviewer-related biases were identified. |
| **Domain 2: study design** | | | |
| *Theoretical framework* | | | |
| 9 | Methodological orientation and Theory | What methodological orientation was stated to underpin the study? e.g. grounded theory, discourse analysis, ethnography, phenomenology, content analysis | Content analysis |
| *Participant selection* | | | |
| 10 | Sampling | How were participants selected? e.g. purposive, convenience, consecutive, snowball | Purposive sampling |
| 11 | Method of approach | How were participants approached? e.g. face-to-face, telephone, mail, email | Email |
| 12 | Sample size | How many participants were in the study? | 37 |
| 13 | Non-participation | How many people refused to participate or dropped out? Reasons? | 106 contacts did not respond to the initial research invitation. 4 provided written consent to participate but dropped out due to a microphone problem (n=1), deferring to another staff member who works in diagnosing and treating patients (n=1) and not responding to the scheduling notification (n=2). |
| *Setting* | | | |
| 14 | Setting of data collection | Where was the data collected? e.g. home, clinic, workplace | Online |
| 15 | Presence of non-participants | Was anyone else present besides the participants and researchers? | No |
| 16 | Description of sample | What are the important characteristics of the sample? e.g. demographic data, date | Interviews were conducted between September 2022 to March 2023. There were 12 clinicians, 10 healthcare leaders, 8 patients and 7 developers. |
| *Data collection* | | | |
| 17 | Interview guide | Were questions, prompts, guides provided by the authors? Was it pilot tested? | All participants were emailed a copy of the semi-structured interview guide in advance of the interview and the interview guide was pilot tested between AL and SH. |
| 18 | Repeat interviews | Were repeat interviews carried out? If yes, how many? | No repeat interviews were carried out. |
| 19 | Audio/visual recording | Did the research use audio or visual recording to collect the data? | Audio recording only |
| 20 | Field notes | Were field notes made during and/or after the interview or focus group? | Yes, after each interview |
| 21 | Duration | What was the duration of the interviews or focus group? | 17-42 minutes (mean 30 minutes) |
| 22 | Data saturation | Was data saturation discussed? | No |
| 23 | Transcripts returned | Were transcripts returned to participants for comment and/or correction? | No |
| **Domain 3: analysis and findings** | | | |
| *Data analysis* | | | |
| 24 | Number of data coders | How many data coders coded the data? | Two, AL and JN |
| 25 | Description of the coding tree | Did authors provide a description of the coding tree? | Yes, in Table 1 |
| 26 | Derivation of themes | Were themes identified in advance or derived from the data? | Identified in advance |
| 27 | Software | What software, if applicable, was used to manage the data? | NVivo (Lumivero, Colorado, USA) |
| 28 | Participant checking | Did participants provide feedback on the findings? | No |
| *Reporting* | | | |
| 29 | Quotations presented | Were participant quotations presented to illustrate the themes / findings? Was each quotation identified? e.g. participant number | Participant quotations were presented to illustrate the findings with each quotation identified by stakeholder type and participant number |
| 30 | Data and findings consistent | Was there consistency between the data presented and the findings? | Yes |
| 31 | Clarity of major themes | Were major themes clearly presented in the findings? | Yes |
| 32 | Clarity of minor themes | Is there a description of diverse cases or discussion of minor themes? | Yes |

JN: Judy Nam, AL: Angelica Ly, SH: Sarita Herse, CL: Chris Lim, MAW: Mary-Anne Williams, FS: Fiona Stapleton
