# Supplementary material for: Stakeholder Perspectives of Implementation Barriers of Artificial Intelligence in Eye Care: A qualitative framework-based study
Source: Ophthalmic Physiol Opt. 2026 Apr 13;46(3):449–58. doi: 10.1007/s44402-026-00080-w (PMC13369640; doi:10.1007/s44402-026-00080-w)
Supplement: Supplementary file 2 [file 44402_2026_80_MOESM2_ESM.docx]

Supplementary file 2. Other barriers identified by stakeholders.

| **CFIR Domain** | **CFIR Sub-Domain** | | **Specific Barriers** | **Stakeholder Example(s)** |
| --- | --- | --- | --- | --- |
| **Innovation** | Innovation Source | | Limited **credibility** of the innovation source | “If you don’t have the knowledge or the information about the technology…people will feel uncomfortable, unfamiliar about using it because there might be certain myths and misconceptions out there…That can certainly be a barrier…the knowledge, the source of credibility behind the technology.” – D5 |
|  | Innovation Adaptability | | **Interoperability**: Limited capacity to work with different imaging instruments, algorithms, and software. | “All just very different in terms of the ability to utilise a specific dataset and translocate that to another machine…So I guess the heterogeneity of the machines available may also limit some of the usefulness.” – C1  “Challenge of whether or not you’re building a one-size-fits all AI tool.” – L5 |
|  | Innovation Complexity | | Nature of ocular imaging being **difficult to acquire** clear images | “False negative cases…because they couldn’t get a clear…good quality image that can run by the artificial intelligence.“ – D4 |
| **Outer Setting** | Partnerships & Connections | | Lack of **partnerships and connections** to support utilisation | “A lot of patients get referred and they think they can probably be seen and treated within the [public] hospital, but they can’t. And that is a barrier…public access to treatment in a lot of cases, and then probably exhaustion to follow up.” – C8 |
|  | External Pressure | | **Misaligned priorities** of different stakeholders | “Sometimes because there are multiple stakeholders in these developments, our purpose may or may not be completely consistent with someone else’s purpose. So, navigating around what the objectives are of the creators, I think, might be a barrier of getting people on the same page.” – L5 |
| **Inner Setting** | Access To Knowledge & Information | | Optometrists’ **lack of training** in being able to use the AI recommendations | “I think technology is great, but…as clinicians need to get better at…having the right tools to be able to actually deliver that information in a way that’s actually going to do something for the patient.” – D3 |
| **Individuals** | Innovation Deliverers | Need | **Optometrists’ fear of job loss** | “Optometrists are going to feel that their jobs are being taken. So that’s the first initial barrier we’re going to have to overcome. So, it’s going to have to be portrayed to the profession as increasing the slice of their pie, not taking away a slice of their pie.” – L4 |
|  |  | Capability | Fear that **professional judgement of clinicians** will be diminished | “I think that there is a little risk that the professional judgement of the practitioner could be diminished. And I think clinicians just see that as a risk.” – L1 |
|  |  | Motivation | **Lack of trust** in the technology itself | “I guess the adoption by clinicians might depend on the willingness to trust the robustness of the algorithm.” – D2 |
|  | Innovation Recipients | Motivation | Lack of individual **patient awareness** regarding the need for eye care | “If you were engaged in a dialogue…then the tendency is to…go and see the doctor for the annual checkup or whatever…Otherwise, you won’t think about it. – P7 |
| **Implementation Process** | Assessing Needs – Innovation Deliverers | | **Training and knowledge** of innovation deliverers, persistent need to retrain | “Training to use the machines properly would be a barrier. And, you know, keeping staff trained and updated on this equipment. As the equipment gets updated, then that would be a barrier you need to keep training.” – L8 |
